# Modified jaw thrust I-gel insertion technique in adults: a case series

**DOI:** 10.1186/s13256-022-03482-9

**Published:** 2022-07-05

**Authors:** Dileep Kumar

**Affiliations:** Department of Anesthesia, Aga Khan University, Stadium Road, P.O. Box 3500, Karachi, Pakistan

**Keywords:** I-gel, General anesthesia, I-gel insertion technique, Laryngospasm

## Abstract

**Background:**

The I-gel is a second-generation supraglottic airway device that is built with a noninflatable elliptical gel material cuff and has a wide semirigid stem. The I-gel supralaryngeal seal has shown promising efficacy for both spontaneous and controlled ventilation under general anesthesia. The recommended, standard I-gel insertion technique is relatively challenging due to its shape and cuff size. Usually, the I-gel becomes entrapped at the oral cavity and requires excessive force to negotiate across the oropharynx, resulting in insertion resistance, tongue obstruction, insertion failure, and intraoral trauma. This case series evaluated a modified jaw thrust I-gel insertion technique because it is claimed to allow smooth and atraumatic I-gel placement in adults.

**Case presentation:**

In this case series, ten male and female Indo-Aryan group of Asian patients aged 18–60 years were recruited for I-gel device placement through a modified jaw thrust technique for short to intermediate surgical duration in below-umbilical surgical procedures. Patient consent was obtained, and baseline vital signs such as electrocardiogram, noninvasive blood pressure, and peripheral oxygen saturation readings were recorded. Following preoxygenation, propofol 2 mg/kg was administered for anesthesia induction and nalbuphine 0.1 mg/kg for analgesia. In all patients, an I-gel was placed by the modified jaw thrust technique. The patient’s demographics, number of attempts, I-gel insertion resistance, and insertion time duration were recorded.

**Conclusion:**

The findings showed a 100% first-attempt insertion rate along with negligible insertion resistance and convincing insertion time duration with modified jaw thrust I-gel insertion technique. However, a blood-stained I-gel was observed in one male patient at the time of removal. The patient’s demographics such as age, weight, American Society of Anesthesiologists status, and surgical and anesthesia duration were found not to be significant. The modified jaw thrust I-gel insertion technique could be considered as an alternative in adults when difficulty is encountered with the standard I-gel insertion technique.

## Background

The I-gel is a second-generation supraglottic airway device that is built with a noninflatable elliptical gel material cuff and has a wide semirigid stem. The design and placement of the I-gel are almost similar to those of the laryngeal mask airway [1]. The I-gel supralaryngeal seal has shown promising efficacy for both spontaneous and controlled ventilation under general anesthesia [2]. The manufacturer’s recommended standard I-gel insertion technique is relatively challenging due to its shape and material, usually causing insertion resistance, tongue obstruction, insertion failure, and intraoral trauma [3]. Various authors have recommended alternative I-gel insertion techniques such as tongue stabilization [4], rotational I-gel insertion [5], and triple I-gel maneuvers [6]. The author has also published a modified jaw thrust I-gel insertion technique [3] as a letter to the editor. This case series of ten patients describes the modified jaw thrust I-gel insertion technique in patients requiring a supraglottic airway device for 15–120 minute duration of below-umbilical surgical procedures.

## Case series

In this case series, ten male and female Indo-Aryan group of Asian patients aged 18–60 years scheduled for elective short to intermediate surgical procedures were recruited for the modified jaw thrust I-gel insertion technique. Pregnant patients as well as those with Mallampati score III and IV, anticipated difficult airway, body mass index (BMI) greater than 30 kg/m^2^, and known history of acid peptic disease were excluded. I-gel insertion was performed by an experienced (more than 1000 placements) anesthesiologist at Aga Khan University Hospital. This case series is part of an ongoing randomized control trial. Patient consent was obtained, and baseline vital signs such as electrocardiogram, noninvasive blood pressure, and peripheral oxygen saturation readings were recorded. Following preoxygenation, propofol 2 mg/kg was administered for anesthesia induction and nalbuphine 0.1 mg/kg for analgesia. The I-gel preinsertion preparation was confirmed, and anesthetic depth was confirmed by jaw relaxation and unresponsiveness of eyelash reflexes. A size 3 I-gel was used for patients weighing less than 50 kg, and size 4 for patients with weight of 50–90 kg. Before applying the modified jaw thrust technique, patients were positioned in sniffing the morning air position with extension of the atlanto-occipital joint along with slight flexion of neck, and the chin was gently pressed down. The I-gel was firmly grasped by the operator at the level of the integral bite block mark, and insertion was initiated with the I-gel cuff outlet facing toward the patient’s chin. The soft tip was inserted into the mouth in a direction toward the hard palate and gently slid to park in the oral cavity. At this stage, both of the operator’s hands were repositioned to thrust the jaw by lifting the angle of the mandibles with the little fingers. Other fingers were used to stabilize the jaw, and force was applied with both thumbs to slide the parked I-gel into the hypopharynx into its final placement position [3]. Successful I-gel insertion was confirmed by capnographic tracing, adequate filling of reservoir bag in spontaneously breathing patients, and appropriate chest expansion on gentle hand ventilation in apneic patients. Anesthesia was maintained with isoflurane minimum alveolar concentration of 1.2, and oxygen and nitrous oxide ratio of 40%/60%. The I-gel was removed in all patients in deep anesthesia at completion of the surgical procedure. Patients’ demographic variables, anesthesia and surgical durations, I-gel insertion attempts, I-gel insertion resistance, I-gel placement time duration, and insertion complications such as laryngospasm, hypoxemia, and airway trauma (blood-stained I-gel at removal) were assessed in all ten cases. The rescue technique was the standard I-gel insertion technique after three consecutive failure attempts with the modified jaw thrust technique.

## Case discussion and conclusion

Supraglottic airway devices (SADs) have an established role in anesthesia practice, as well as for rapid-response care givers. To improve patient safety, several modifications and refinements in their structure and insertion technique have been redefined. However, several features such as minimal gas flow resistance, adequate airway seal pressure for both spontaneous and positive pressure ventilation, compatibility with surgical procedures (laparoscopy, pregnancy, and in obese patients), negligible pulmonary aspiration risk, better first-attempt insertion rates for experienced, novice, and prehospital care staff, and manageable complication rates still require attention [1]. The I-gel can deliver adequate oropharyngeal leak pressure and gastric access with comparable insertion speed and successful insertion attempts. However, the shape and cuff size of the I-gel mean that it usually becomes entrapped at the oral cavity and requires force to pass through the oropharynx, resulting in insertion trauma. The author proposed a modified jaw thrust I-gel insertion technique, which was found to enable smooth intraoral I-gel negotiation by providing adequate space in the oropharynx [3].

This case series recruited five male and five female patients who underwent general anesthesia. Patients were similar in age, weight, ASA status, anesthesia, and surgical duration (Table 1). A 100% first-attempt insertion rate was observed, along with negligible insertion resistance and convinciing insertion time duration (Table 2). The I-gel insertion time duration was noted to be 11.1 ± 4.25 seconds, and a blood-stained I-gel was observed in one male patient (Table 2). Barman *et al.* [7] tested a two-person jaw thrust I-gel insertion technique and found a first-attempt insertion rate of 92.5%, while another author reported a rate of 96% with reverse I-gel insertion versus 86% with the standard technique [5]. The I-gel insertion time duration was superior (11.1 ± 4.25 seconds) in this case series compared with the two-person jaw thrust technique (14.025 ± 1.99 seconds), reverse I-gel insertion technique (17.5 ± 6.5 s), or standard technique (20.8 ± 5.9 seconds). The incidence of trauma (blood-stained I-gel) in our study was 10%, compared with 5% in the two-person jaw thrust technique. The modified jaw thrust I-gel insertion works by creating an adequate intraoral space for the I-gel to negotiate the hypopharynx smoothly along with stabilization of the device in the center of the oral cavity. It has the added advantage of one-person operator harmonization to thrust the jaw compared with requiring the assistance of a second person to facilitate the jaw thrust in the two-person jaw thrust insertion technique. A randomized control trial with a larger number of patients will confirm the reliability of this technique and help to establish its role in clinical practice. This case series found that the modified jaw thrust I-gel insertion technique is practical and facilitates intraoral negotiation with minimal resistance, an insignificant complication rate, and an excellent first-pass insertion rate. This technique could be considered in adults as an alternative when difficulty is encountered with the standard I-gel insertion technique.

**Table 1:** Patients’ demographics and anesthesia and surgical durations

Age (years)	39.90 ± 18.75
Weight (kg)	68.50 ± 14.77
Height (cm)	161.70 ± 8.26
Heart rate (beats per minute)	79.30 ± 11.48
Preoperative systolic blood pressure (mmHg)	129.2 ± 18.47
Preoperative diastolic blood pressure (mmHg)	75.30 ± 10.21
Peripheral oxygen saturation (%)	97.70 ± 0.82
Gender	
Male	5
Female	5
ASA	
I	4
II	6
Mallampatti grade	
I	3
II	7
Duration of surgery (minutes)	67.6 ± 41.66 (range 11–121)
Duration of anesthesia (minutes)	100.8 ± 59.05 (range 21–213)

kg: Kilogram, cm: Centimeters, mmHg: Millimeter of mercury, %: Percentage, ASA: American Society of Anesthesiologists

**Table 2:** I-GEL: number of insertion attempts, insertion duration, insertion resistance, and insertion complications

Number of attempts at I-gel insertion	
First-attempt insertion	10
Second-attempt insertion	0
Third-attempt insertion	0
I-gel insertion time duration (seconds)	11.1 ± 4.25 (range 4–18)
I-gel insertion resistance	
Mild	4
Moderate	1
Laryngospasm	0
Hypoxemia	0
Airway trauma (blood-stained I-gel on removal)	1

## Data Availability

The datasets used and/or analyzed during the current study are available from the corresponding author on reasonable request.
